# The Effect of Acute Aerobic Exercise on Redox Homeostasis and Mitochondrial Function of Rat White Adipose Tissue

**DOI:** 10.1155/2021/4593496

**Published:** 2021-01-31

**Authors:** Leonardo Matta, Túlio S. Fonseca, Caroline C. Faria, Niedson Correia Lima-Junior, Dahienne F. De Oliveira, Leonardo Maciel, Luiz F. Boa, Ana Paola T. R. Pierucci, Andrea C. F. Ferreira, José H. M. Nascimento, Denise P. Carvalho, Rodrigo S. Fortunato

**Affiliations:** ^1^Carlos Chagas Filho Institute of Biophysics, Federal University of Rio de Janeiro, 21941-590, Brazil; ^2^Josué de Castro Institute of Nutrition, Federal University of Rio de Janeiro, Brazil; ^3^NUMPEX, Duque de Caxias Campus, Federal University of Rio de Janeiro, Brazil

## Abstract

Physical exercise is characterized by an increase in physical and metabolic demand in face of physical stress. It is reported that a single exercise session induces physiological responses through redox signaling to increase cellular function and energy support in diverse organs. However, little is known about the effect of a single bout of exercise on the redox homeostasis and cytoprotective gene expression of white adipose tissue (WAT). Thus, we aimed at evaluating the effects of acute aerobic exercise on WAT redox homeostasis, mitochondrial metabolism, and cytoprotective genic response. Male Wistar rats were submitted to a single moderate-high running session (treadmill) and were divided into five groups: control (CTRL, without exercise), and euthanized immediately (0 h), 30 min, 1 hour, or 2 hours after the end of the exercise session. NADPH oxidase activity was higher in 0 h and 30 min groups when compared to CTRL group. Extramitochondrial ROS production was higher in 0 h group in comparison to CTRL and 2 h groups. Mitochondrial respiration in phosphorylative state increased in 0 h group when compared to CTRL, 30 min, 1, and 2 h groups. On the other hand, mitochondrial ATP production was lower in 0 h in comparison to 30 min group, increasing in 1 and 2 h groups when compared to CTRL and 0 h groups. CAT activity was lower in all exercised groups when compared to CTRL. Regarding oxidative stress biomarkers, we observed a decrease in reduced thiol content in 0 h group compared to CTRL and 2 h groups, and higher levels of protein carbonylation in 0 and 30 min groups in comparison to the other groups. The levels returned to basal condition in 2 h group. Furthermore, aerobic exercise increased NRF2, GPX2, HMOX1, SOD1, and CAT mRNA levels. Taken together, our results suggest that one session of aerobic exercise can induce a transient prooxidative state in WAT, followed by an increase in antioxidant and cytoprotective gene expression.

## 1. Introduction

Physical exercise is characterized by an increase in physical and metabolic demands in face of physical stress, in a programmed, periodized, and progressive way that allows several adaptations in organic systems [1]. An acute exercise session disturbs the body's homeostasis, leading to thermal, metabolic, and oxidative stress. Several biochemical messengers are released due to these homeostatic perturbations, including Ca^2+^, growth factors, cytokines, and reactive oxygen species (ROS). These messengers will stimulate a wide range of signaling pathways that will mediate acute and chronic responses to exercise [2]. Understanding acute responses to exercise is important for understanding how exercise conditions the body and induces long-term adaptations.

In response to exercise, an increase in skeletal muscle ROS generation is observed, mainly due to the increase of NADPH oxidase (NOX) and xanthine oxidase (XO) activities [3–5]. The transient increase in skeletal muscle ROS induced by acute exercise activates redox-sensitive signaling pathways that are involved in many physiological responses to exercise and subsequent muscular adaptations, including the increment of antioxidant capacity [6]. Chronically, repetitive bouts of exercise improve antioxidant factor 2 (Nrf2) a transcription factor responsible to orchestrate the antioxidant defense, regulating the expression of more than 200 cytoprotective genes [7]. Interestingly, increased ROS generation and biomolecules oxidation after acute exercise are found not only in skeletal muscle but also in other organs [8, 9]. Furthermore, some pieces of evidence show that physical exercise is able to activate Nrf2/ARE (antioxidant response element) signaling and to increase antioxidant defense beyond skeletal muscle. Muthusamy et al. (2012) showed that acute exercise was able to activate Nrf2/ARE signaling and the subsequent enhancement of antioxidant defense in mouse hearts [10]. Tsou et al. (2015) evaluated if a treadmill exercise protects the nigrostriatal dopaminergic neurons by inducing Nrf2 antioxidant system in 1-methyl-4-phenylpyridine- (MPP+-) induced Parkinsonian rat model. This study showed that treadmill exercise for 4 weeks induced upregulation of Nrf2 and gamma-glutamylcysteine ligase (*γ*GCLC) expression and also prevented the MPP+-induced downregulation of Nrf2/*γ*GCLC/glutathione and nigrostriatal dopaminergic neurodegeneration [11]. Moreover, regular exercise has also been shown to activate Nrf2 signaling in other tissues as liver [12], kidney [13], prostate [14], and testis [15].

White adipose tissue (WAT) is a key metabolic tissue, not only important for regulating energy availability during and after exercise but also by performing an endocrine role through the secretion of adipokines, that affects the metabolism and function of different organs [16]. Physical exercise increases WAT blood flow and adrenergic stimulation that results in higher lipolysis and fatty acid oxidation rates [17, 18]. Recently, Townsend et al. (2020) showed in a genetically modified mouse model of reduced mitochondrial ROS emission (mitochondrial catalase overexpression—MCAT) that exercise-induced ROS regulates molecular responses in WAT. In wild-type mice, acute exercise induced a transient increase in *Pdk4* and phosphoenolpyruvate carboxykinase (*Pck1*) mRNA in subcutaneous inguinal WAT and epididymal WAT depots, which was not observed in MCAT animals. These results suggest that ROS is involved in WAT physiological responses to physical exercise [19]. Sakurai et al. (2009) reported that rats submitted to 9 weeks of aerobic exercise had a higher superoxide dismutase 2 expression in epididymal WAT, while the expression of the ROS-producing enzyme NOX2 and lipid peroxidation was decreased in epididymal and retroperitoneal WAT [20]. Moreover, 8 weeks of exercise training increased SOD2 and catalase protein levels and decreased lipid peroxidation in WAT of male old rats [21]. These effects of chronic physical exercise on redox homeostasis seem to be an adaptation to the acute exposure of ROS elicited by each single bout of exercise as observed in several tissues.

The studies in the literature suggest through indirect evidences that ROS could mediate the effects of acute exercise on WAT. The sources of ROS stimulated by acute exercise in WAT, as well as the modulation of redox-sensitive responses, are not completely understood. Thus, we aimed at evaluating the effect of acute aerobic exercise on WAT redox homeostasis, as well as the expression of genes related to the activation of NRF2-KEAP1-ARE pathway in male adult Wistar rats.

## 2. Material and Methods

### 2.1. Experimental Model

Adult male Wistar rats weighing 400–450 g with 18 weeks age were maintained in an animal room with controlled lighting (12-h light-dark cycle) and temperature (23–24°C) with free access to standard rat chow and drinking water. The Institutional Committee for Use of Animals in Research approved the study (Protocol n°: 132/18), and the procedures were in compliance with the International Guiding Principles for Biomedical Research Involving Animals of the Council for International Organizations of Medical Sciences (Geneva, Switzerland). The animals were divided in control group (CTRL)—without exercise and four groups (n =6/group) euthanized at different time points after the exercise session: euthanized immediately after the exercise session (0 h) and euthanized 30 minutes (30 min), 1 hour (1 h), or 2 hours after (2 h).

Prior to acute exercise, the animals of experimental groups were adapted to the treadmill for 1 week, with an average speed of 10 m/min for 10 minutes for 5 days. After the adaptation, the incremental test of maximum effort was performed with an initial speed of 6 m/min, which was increased 3 m/min every 3 minutes with fixed inclination at 10°. The test was carried out until animal's exhaustion (when the animal stayed in the steel grid despite increasing shock stimuli) adapted from Bacurau et al. (2016) [22] and according to previous studies by our group [23]. The average maximum speed reached was 34 ± 4 cm/s, and the average effort time was 30 ± 5 minutes. For the acute exercise session, the animals run between 65% and 75% of their maximum speed (obtained in the maximum effort test) for 20 minutes that corresponds to moderate-high intensity aerobic exercise. After that, the animals were euthanized by decapitation, and the retroperitoneal WAT was extracted and stored at -80°C until further analyses (maximum four weeks of storage).

### 2.2. Lactate Measurement Assay

Blood samples were collected from the tail vein immediately after the maximum effort test and placed in a tube with 25% sodium fluoride (NaF). The plasma was obtained by centrifugation of blood at 2,000 x g in room temperature for 15 minutes. Lactate levels were measured in plasma samples using a commercially available Bioclin kit (Quibasa, Brazil), in accordance with the manufacturers' instructions. The measurement is based on the reaction of lactic dehydrogenase (LDH) catalyzing the oxidation of L-Lactate to Pyruvate, with a consequent reduction of NAD^+^ to NADH. NADH formation was measured by spectrophotometry at 340 mm in a microplate reader (Victor X4; PerkinElmer). The values were obtained through the product of the sample absorbance by the calibration factor (concentration of the standard curve × standard absorbance), obtaining a concentration in mg/dL. The values were presented in mmol/L (lactate concentration (mg/dL) × 0.1109) [24].

### 2.3. NADPH Oxidase Activity

NADPH oxidase activity was quantified in the retroperitoneal WAT using the Amplex Red/Horseradish Peroxidase (HRP) Assay (Molecular Probes, Invitrogen). Retroperitoneal WAT (700 mg) was homogenized in 50 mM sodium phosphate buffer, pH 7.2, containing 0.25 M sucrose, 0.5 mM DTT, 1 mM EGTA, 5 mg/mL aprotinin, and 34.8 mg/mL PMSF. The homogenate was centrifuged at 700 x g for 10 minutes. After centrifugation, the supernatant lipid residue was discharged and the intermediate phase was collected and centrifuged at 100,000 x g for 35 minutes at 4°C. The pellet was resuspended in 0.5 mL of 50 mM sodium phosphate buffer, pH 7.2, containing 0.25 M sucrose, 2 mM MgCl_2_, 5 mg/mL aprotinin, and 34.8 mg/mL phenylmethanesulfonyl fluoride (PMSF) and stored at –80°C until H_2_O_2_ generation measurements.

The microsomal fraction was incubated in 150 mM sodium phosphate buffer (pH 7.4) containing SOD (100 U/ml; Sigma), HRP (0.5 U/mL, Roche, Indianapolis, IN), NADPH (1 mM) and Amplex Red (50 *μ*M; Molecular Probes, Eugene, OR), and the fluorescence was immediately measured in a microplate reader (Victor X4; PerkinElmer, Norwalk, CT) at 30°C, using an excitation wavelength of 530 nm and an emission wavelength of 595 nm during 1 hour. The enzymatic activity was expressed as nanomoles of H_2_O_2_ per hour per milligram of protein (nmol·h^−1^·mg^−1^) [25]. Protein concentration was determined by the Bradford assay [26].

### 2.4. Mitochondria Isolation and Measurement of Mitochondrial Function

Mitochondria isolation was performed immediately after euthanasia by differential centrifugation according to the modified protocol of Maciel et al. (2020) [27]. The retroperitoneal WAT [5% tissue weight/volume (*w*/*v*)] was placed in ice-cold mitochondria-isolation buffer containing (in mmol·L^−1^) 250 sucrose, 10 HEPES, 1 ethylene glycol tetra acetic acid (EGTA), and pH 7.4 without bovine serum albumin (BSA). The tissue was minced carefully using scissors. Next, the minced tissue was homogenized with a tissue homogenizer (Ultra-Turrax) using two 10-sec treatments at a shaft rotation rate of 6,500 rpm. This homogenate was further homogenized using a Teflon pestle. The homogenate was centrifuged at 700 x g for 10 min at 4°C. The supernatant was collected and diluted in a cold isolation buffer containing Percoll (20%), and centrifuged at 14,000 x g for 10 min at 4° C. This procedure was repeated with isolation buffer without BSA containing Percoll (10%). The resulting pellet was resuspended in isolation buffer without BSA and Percoll and centrifuged at 10,000 x g for 5 min at 4°C. This procedure was repeated with isolation buffer without BSA and Percoll, and the pellet was resuspended in mitochondria-isolation buffer. The protein concentration of the isolated pellet was verified using a protein assay (Lowry method, Biorad, Hercules, CA, USA) by comparison to a BSA standard (Thermo Scientific, Waltham, MA, USA).

#### 2.4.1. Mitochondrial Oxygen Consumption

Mitochondrial respiration was measured with a Clark-type electrode (Strathkelvin, Glasgow, United Kingdom) at 37°C during magnetic stirring in respiration buffer containing in mmol·L^−1^: 125 KCl, 10 MOPS, 2 MgCl_2_, 5 KH_2_PO_4_, 0.2 EGTA with pyruvate (5 mmol·L^−1^), and malate (5 mmol·L^−1^) as substrates for complex I. The oxygen electrode was calibrated using a solubility coefficient of 217 nmol O_2_/mL at 37°C. For the measurement of complex I respiration, mitochondria (corresponding to a mitochondrial protein amount of 200 *μ*g) were added to 1 mL of incubation buffer. After 2 min of incubation, 1 mmol·L^−1^ ADP was added, and ADP-stimulated respiration was measured for 2 min. Mitochondria were used to either measure complex IV respiration, and maximal uncoupled oxygen uptake in the respiration chamber or the respiration buffer containing mitochondria was taken from the respiration chamber to measure ATP production and extramitochondrial ROS concentration. Complex IV respiration was stimulated by adding N,N,N,N′-tetramethyl-p-phenylenediamine (TMPD, 300 *μ*mol·L^−1^) plus ascorbate (3 *μ*mol·L^−1^). Maximal uncoupled oxygen uptake was measured in the presence of 30 nmol·L^−1^ carbonyl cyanide-p-trifluoromethoxyphenyl-hydrazone (FCCP) [27].

#### 2.4.2. Mitochondrial ATP Production

After the measurement of ADP-stimulated respiration, the incubation buffer containing mitochondria was taken from the respiration chamber and immediately supplemented with the ATP Assay Mix (diluted 1 : 5) (Sigma, Aldrich). Mitochondrial ATP production after each respiration measurement was determined immediately and compared with ATP standards using a 96-well white plate and a spectrofluorometer (SpectraMax® M3, Molecular Devices, EUA) at 560 nm emission wavelength [27].

#### 2.4.3. Extramitochondrial ROS Concentration

The Amplex Red Hydrogen Peroxide Assay Kit (Life Technologies, Carlsbad, CA, USA) was used to determine extramitochondrial ROS production. Amplex Red reacts at 1 : 1 stoichiometry with peroxides under catalysis by HRP and produces highly fluorescent resorufin. The incubation buffer containing mitochondria was removed from the respiration chamber and immediately supplemented with 50 *μ*mol·L^−1^Amplex UltraRed and 2 U/mL HRP. The supernatant was collected after 120 min of incubation in the dark. Extramitochondrial ROS concentration was determined and compared with H_2_O_2_ standards using a 96-well black plate and a spectrofluorometer (SpectraMax® M3, Molecular Devices, EUA) at 540 nm emission and 580 nm extinction wavelengths [27].

### 2.5. Antioxidant Enzymes Activities

Retroperitoneal WAT was homogenized in 5 mM Tris-HCl buffer (pH 7.4), containing 0.9% NaCl (w/v) and 1 mM EDTA, followed by centrifugation at 750 x g for 10 minutes at 4°C. The supernatant aliquots were stored at -80° C. Total superoxide dismutase (SOD) activity was determined by reduction of cytochrome C at 550 nm [28]. Catalase (CAT) activity was assayed following the method of Aebi (1984) and was expressed as units per milligram of protein (U/mg) [29]. Glutathione peroxidase (GPX) activity was assayed by following NADPH oxidation at 340 nm in the presence of an excess of glutathione reductase, reduced glutathione, and tert-butyl hydroperoxide as substrates and expressed as nmol of oxidized NADPH per milligram of protein (nmol/mg) [30]. Protein concentration was determined by the Bradford assay.

### 2.6. Biomarkers of Oxidative Damage

#### 2.6.1. Reactive Protein Thiol Levels

Reactive protein thiol levels were measured using 5,5′-dithio-bis-(2-nitrobenzoic acid) (DTNB) (Sigma Aldrich). Thiol residues react with DTNB, cleaving the disulfide bond to give 2-nitro-5-thiobenzoate (NTB^−^), which ionizes to the NTB_2_^−^ di-anion in water at neutral and alkaline pH. The NTB_2_^−^ was quantified in a spectrophotometer by measuring the absorbance at 412 nm [31].

#### 2.6.2. Lipid Peroxidation by Western Blotting

Protein samples were mixed with 2x Laemmli Sample Buffer for preparation of samples for SDS PAGE. (BioRad, Hercules, CA) and separated using a 10% Bis-Acrylamide gel at 130 V for 60-120 minutes (BioRad, Hercules, CA). Resolved proteins were then electrophoretically transferred onto nitrocellulose membranes (BioRad, Hercules, CA) at 30 V overnight. After that, to assess protein loading and transfer, membranes were incubated in 0.1% (*w*/*v*) Ponceau S in 5% acetic acid and, then, were digitally photographed by Image Quant LAS 500. The membranes were blocked for 1 h in TBS-T (20 mM Tris-HCl, pH 7.6, 150 mM NaCl, 0.1% Tween) containing 3% bovine serum albumin (BSA). A 1 : 700 dilution of the primary antibody anti-4-HNE (Abcam, Cambridge, UK) was added and stirred overnight at 4°C, followed by 3 washes with 1x TBS-T. Then, the membranes were incubated for 1 h at room temperature with a 1 : 10.000 dilution of HRP-linked anti-mouse secondary antibody (Abcam, Cambridge, UK) in 3% BSA/TBS-T. The bands were visualized with Luminata™ Western HRP (Millipore, Millerica, MA) using Image Quant LAS 4000 (GE Life Science, Boston, EUA). The data were expressed as the densitometric ratio of the 4-HNE column to the total protein in each band obtained by red ponceau staining and normalized by the control group; both measures were obtained using the software Image J [32].

#### 2.6.3. Carbonylated Proteins by 2D OxyBlot

The retroperitoneal WAT homogenate used for antioxidant analysis was denatured and derivatized with a 12% solution of sodium dodecyl sulfate (SDS) and dinitrophenylhydrazine (DNPH) according to the manufacturer's protocol (Millipore). A neutralization solution was used to terminate the derivatization reaction after 15 min. Protein separation was performed using a 12% Bis-Acrylamide gel at 120 V for 60-120 minutes (BioRad), followed by transfer to a nitrocellulose membrane at 25 V overnight. Nonspecific binding sites were blocked with 1x phosphate-buffered saline and Tween 20 (1x PBS-T) and 5% BSA for one hour. A 1 : 500 dilution of the primary antibody (Anti-Rabbit-NDP—Kit Millipore OxyBlot) was added and stirred overnight at 4°C, followed by 3 washes with 1x PBS-T. Then, the membranes were incubated with a 1 : 300 dilution of goat Anti-Rabbit IgG (conjugated with peroxidase) antibody for one hour at room temperature. The bands were visualized with Luminata™ Western HRP (Millipore, Millerica, MA) using BioRad Chemidoc and Image Lab (BioRad, Hercules, CA). The data were expressed as the densitometric ratio of the dinitrophenyl-hydrazone (DNP) bands to the total protein in each band obtained by the endogenous control tag by *β*-actin and normalized by control group [33].

### 2.7. Gene Expression by Real-Time Q-PCR

Total RNA was extracted from retroperitoneal WAT using Dynabeads™ and TissueLyser LT by disruption and homogenization through high-speed shaking of samples in 2 ml microcentrifuge. For RNA extraction, RNeasy Lipid Tissue Mini Kit (Qiagen, USA) was used following the manufacturer's instructions. RNA concentration and purity were determined by measuring the sample's absorbance at 260 and 280 nm with a spectrophotometer (Biomate 3S, Thermo Scientific), and integrity was analyzed by electrophoresis in agarose gel (1%). cDNA was synthesized from 1.2 *μ*g of RNA in a thermocycler (Techne TC-412, UK) using High-Capacity cDNA Reverse Transcription Kit (Invitrogen, USA), according to the manufacturer's instructions. Real-time PCR was performed using EvaGreen (HOT FIREPol EvaGreen HRM mix, Solis BioDyne). The qPCR cycle was set according to the manufacturer's instructions (initial denaturation 95°C for 15 min once; followed by denaturation 95°C for 15 s plus annealing 60°-65°C for 20 s and elongation 72°C for 20 s, repeated 40 times). The *β*-actin gene was used as the housekeeping control gene. Gene expression was analyzed using the 2^-*ΔΔ*Ct^ method [34]. Description of primers used for real-time PCR is referred in Table 1.

### 2.8. Statistical Analysis

The results were expressed as mean ± standard error of the mean (SEM) and analyzed through the statistical program GraphPad Prism 7.0 (San Diego, CA, USA). The D'Agostino and Pearson test was used to verify the normality of the samples of blood lactate levels, followed by the paired *t*-test. Other data sets were tested for normality using the Kolmogorov-Smirnov test followed by the one-way ANOVA analysis of variance with Bonferroni multiple comparisons as posttest.

## 3. Results

### 3.1. Maximal Effort Characterization

**Figure 1:**
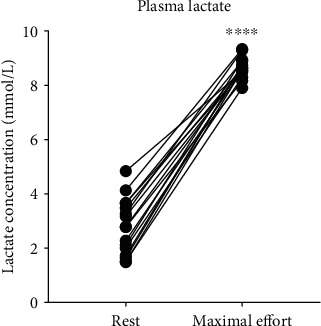
Lactate plasma concentration at rest and maximum postexercise in an incremental test of maximum speed. Lactate levels were measured in plasma by spectrophotometry using the BioClin ® Kit. The data were expressed as individual values (*n* = 16). ^∗∗∗∗^*p* < 0.0001.

In order to validate the maximum effort test, we measured plasma lactate concentration (Figure 1). We observed higher plasma lactate levels after the maximum effort test when compared to its levels before the test (*p* < 0.0001). This result demonstrates that all animals reached the maximum effort level, once plasma lactate was higher than 7 mmol/L, which ensures greater accuracy in the calculation of % of the maximum individual running velocity for the exercise.

### 3.2. Effect of Acute Aerobic Exercise on NADPH Oxidase Activity and Extramitochondrial ROS Production in Retroperitoneal WAT

**Figure 2:**
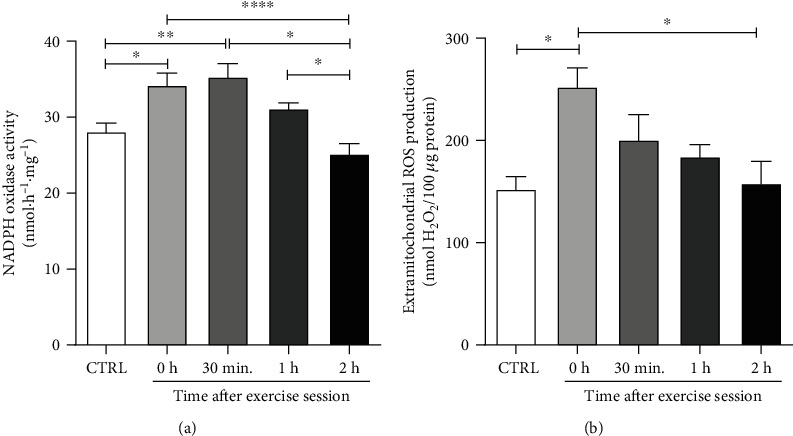
The effects of an acute exercise on the main sources of ROS of retroperitoneal WAT in rats. The animals were submitted to aerobic training on a treadmill for 20 minutes at an intensity of 75% of maximum speed and euthanized at different time points. (a) NADPH oxidase activity was measured by spectrophotometry in microsomal fraction (*n* = 7/group). (b) Generation of mitochondrial H_2_O_2_ measured by spectrophotometry (*n* = 4/group). CTRL: control; 0 h; euthanasia immediately after exercise; 30 min: euthanasia 30 minutes after exercise; 1 h: euthanasia 1 hour after exercise; 2 h: euthanasia 2 hours after exercise. The data were expressed as the mean ± standard error of the mean (*n* = 7/group). ^∗^*p* < 0.05; ^∗∗^*p* < 0.01; ^∗∗∗∗^*p* < 0.0001.

Firstly, we evaluated the effect of acute aerobic exercise in the two main sources of ROS in retroperitoneal WAT: NADPH oxidase enzymes and mitochondria [35]. Regarding NOX activity, a significant increase was observed immediately and 30 minutes after exercise in comparison to CTRL group (CTRL vs. 0 h, *p* < 0.05; CTRL vs. 30 min, *p* < 0.01). However, this effect was transient, since a significant decrease in NOX activity was observed in the 2 h group in comparison to all the other exercised groups (0 h vs. 2 h: *p* < 0.0001; 30 min vs. 2 h: *p* < 0.05; and 1 h vs. 2 h: *p* < 0.05), but not to control (Figure 2(a)). In relation to the extramitochondrial ROS production, we observed a higher production of ROS immediately after the exercise session (0 h) in comparison to the CTRL group (CTRL vs. 0 h: *p* < 0.05). Interestingly, similarly to NOX activity, this effect was transient and returned to basal levels after 2 hours of the exercise session (0 h vs. 2 h: *p* < 0.05) (Figure 2(b)). These findings indicate that acute exercise stimulates a transient increase in retroperitoneal WAT ROS production mediated by NOX enzymes and mitochondria.

### 3.3. Acute Exercise Effects on Mitochondrial Respiration and ATP Production

**Figure 3:**
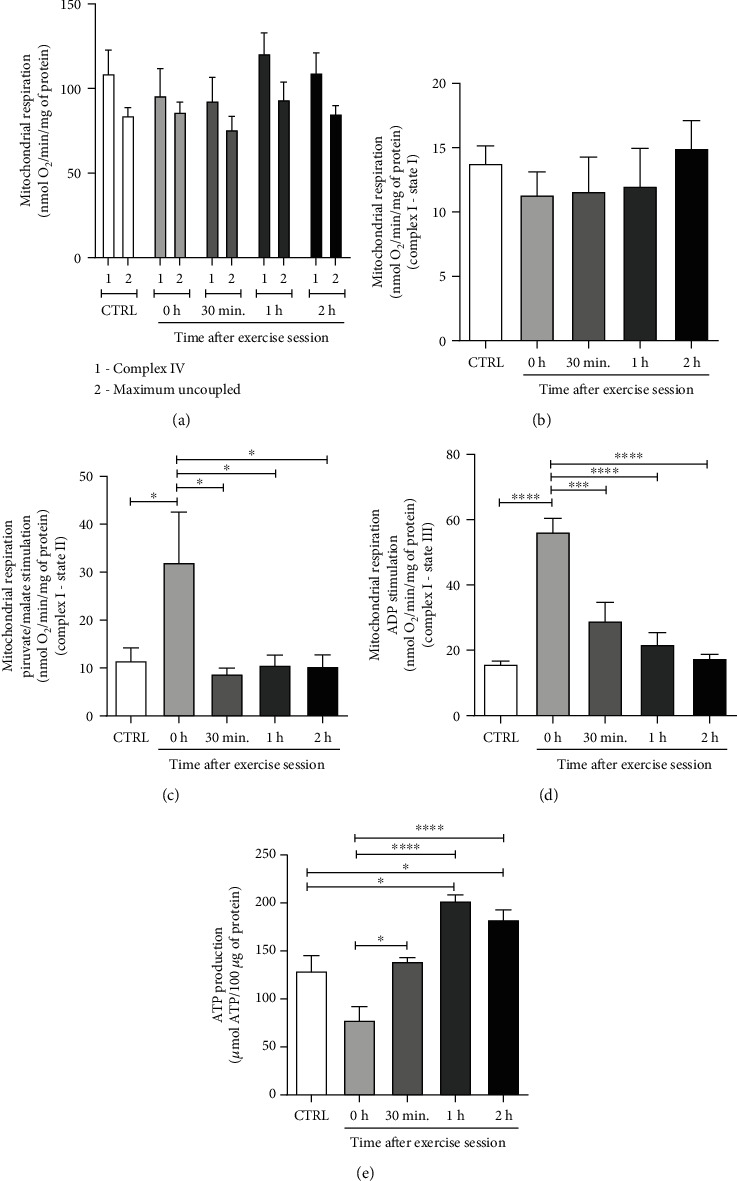
Effect of an acute exercise session on mitochondrial respiration and ATP production levels on retroperitoneal WAT in rats. (a) Mitochondrial oxygen consumption at baseline and maximum levels, FCCP was added at 30 *μ*mol to assess the maximum consumption of O_2_. (b) Mitochondrial respiration at rest, free of complex I activation substrate. (c) Mitochondrial respiration in state II of complex I, stimulated by the addition of pyruvate (5 mmol·L^−1^) and malate (5 mmol·L^−1^). (d) Mitochondrial respiration in state III of complex I (oxidative phosphorylation) stimulated by the addition of ADP (1 mmol·L^−1^) for 2 minutes. (e) ATP production by spectrophotometry. CTRL: control; 0 h; euthanasia immediately after exercise; 30 min: euthanasia 30 minutes after exercise; 1 h: euthanasia 1 hour after exercise; 2 h: euthanasia 2 hours after exercise. The data were expressed as the mean ± standard error of the mean (*n* = 5). ^∗^*p* < 0.05; ^∗∗^*p* < 0.01; ^∗∗∗^*p* < 0.001; ^∗∗∗∗^*p* < 0.0001.

Mitochondria are one of the main sources of ROS in adipose tissue [36]. Since extramitochondrial ROS production was transiently increased after exercise, we analyzed mitochondrial respiration and ATP production, in order to evaluate mitochondrial function. Our results showed that mitochondrial complex IV respiration and maximal oxygen uptake of uncoupled mitochondria were not different among groups, reflecting an equal loading of viable mitochondria in all experiments (Figure 3(a)). There were also no significant changes in mitochondrial oxygen consumption, specifically, in complex I activity, in state I (Figure 3(b)). However, we observed that in state II of complex I, the 0 h group had a higher consumption of O_2_ in relation to all the other groups (0 h vs. CTRL, *p* < 0.01; 0 h vs. 30 min, *p* < 0.004; vs. 1 h, *p* < 0.009 and vs. 2 h, *p* < 0.001) (Figure 3(c)). In addition, when analyzing state III of complex I, we found that immediately after exercise (0 h) O_2_ consumption was significantly higher compared to the other groups (0 h vs. CTRL, *p* < 0.0001; vs. 30 min, *p* < 0.001; vs. 1 h and 2 h: *p* < 0.0001) (Figure 3(d)). Interestingly, we found that despite the high oxygen mitochondrial consumption in the phosphorylative state of complex I in 0 h group, it was not accompanied by an increase in ATP production. Instead, ATP production had a tendency to reduce immediately after exercise when compared to control group (CTRL vs. 0 h, *p* = 0.0518), thus suggesting that the oxygen consumed by mitochondria is deviated to ROS production instead of ATP synthesis. In 30 min group, it was demonstrated that ATP production increased significantly compared to 0 h (0 h vs. 30 min: *p* < 0.05). ATP production of 1 h group was higher than CTRL (CTRL vs. 1 h, *p* < 0.05) and 0 h groups (0 h vs. 1 h, *p* < 0.0001), as well as 2 h group (CTRL vs. 2 h, *p* < 0.05: CTRL vs. 0 h, *p* < 0.0001). No differences were found between 1 h and 2 h groups (Figure 3(e)). These results suggest a negative relationship between ROS and ATP production in mitochondria.

### 3.4. Antioxidant Enzymes Activities

**Figure 4:**
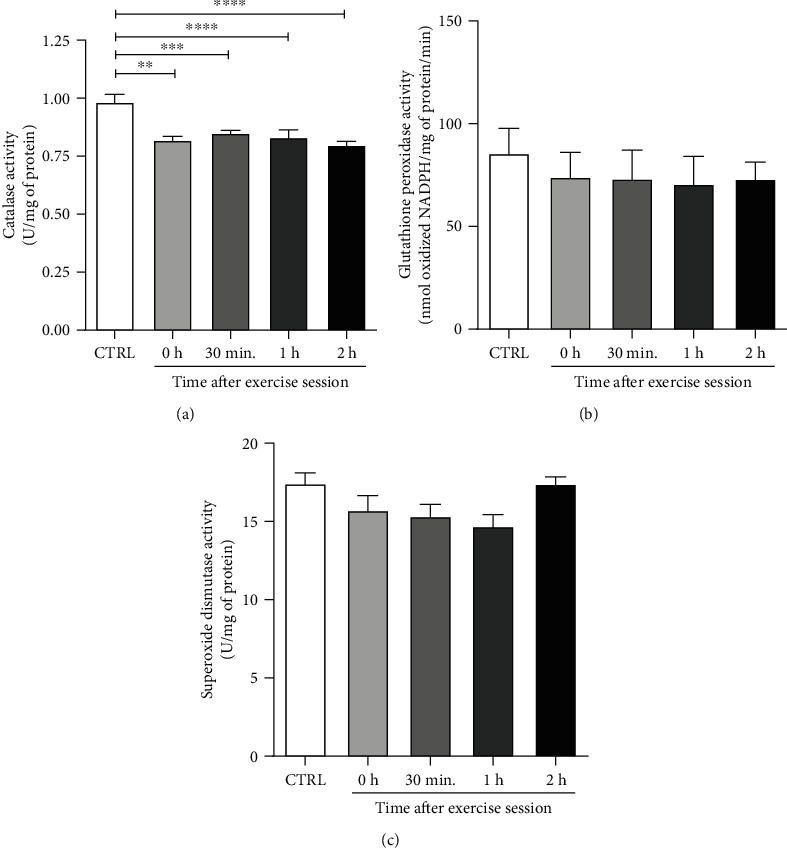
Effect of an acute exercise session on the antioxidant enzymatic activity of retroperitoneal WAT in rats. (a) Catalase, (b) GPX, and (c) SOD activities were measured in retroperitoneal WAT homogenates by spectrophotometry. CTRL: control; 0 h; euthanasia immediately after exercise; 30 min: euthanasia 30 minutes after exercise; 1 h: euthanasia 1 hour after exercise; 2 h: euthanasia 2 hours after exercise. The data were expressed as the mean ± standard error of the mean (*n* = 7). ^∗∗^*p* < 0.01; ^∗∗∗^*p* < 0.001; ^∗∗∗∗^*p* < 0.0001.

ROS availability in a given tissue depends on their production and detoxification rates. Since our results demonstrate an increase in ROS production after exercise, we decided to evaluate the activity of the antioxidant enzymes CAT, SOD, and GPX. CAT activity was lower in all exercised groups in comparison to control (CTRL vs. 0 h, *p* < 0.001; vs. 30 min, *p* < 0.01; vs. 1 h, *p* < 0.001; vs. 2 h, *p* < 0.0001) (Figure 4(a)). However, no differences were observed among groups for GPX (Figure 4(b)) and SOD (Figure 4(c)) activities.

### 3.5. Biomarkers of Oxidative Damage

**Figure 5:**
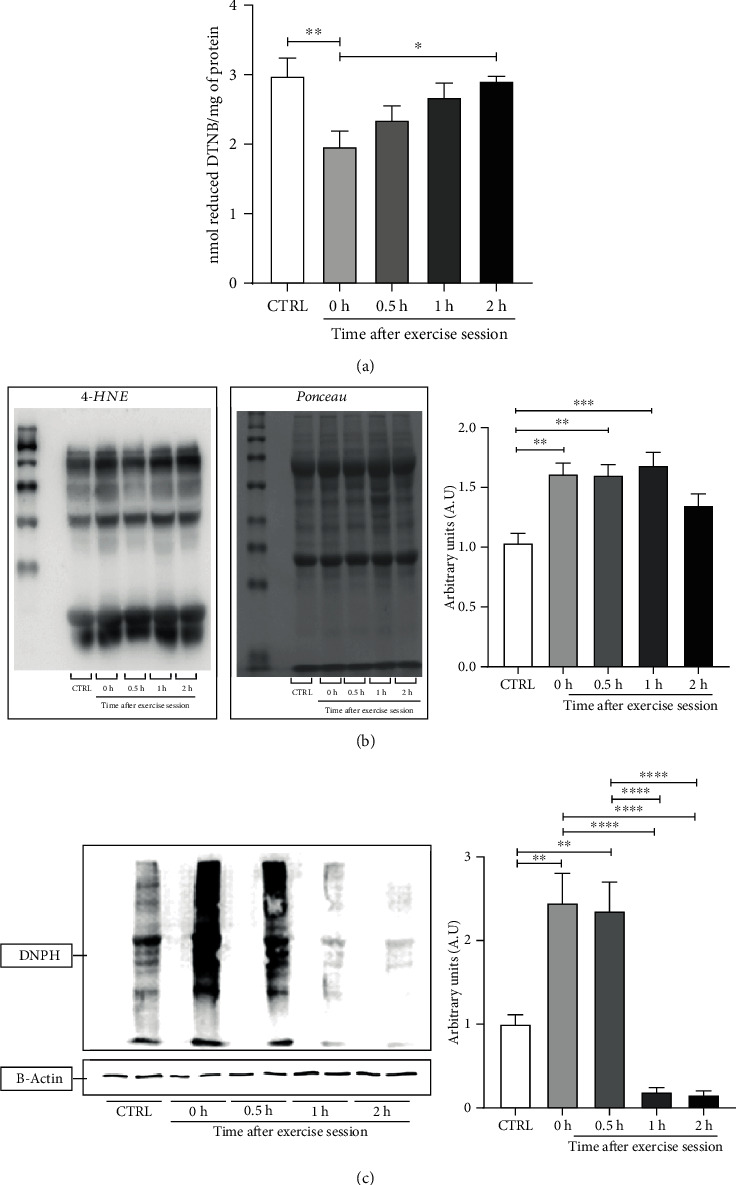
Effect of an acute exercise session on biomarkers of oxidative damage in the retroperitoneal WAT of rats. (a) Levels of reduced thiol content. Total sulfhydryl groups were measured in homogenates of retroperitoneal WAT by spectrophotometry. DTNB: 5,5′-dithiobis-(2-nitrobenzoic acid) (*n* = 7/group). (b) Levels of lipid peroxidation, representative result of a membrane incubated with anti-4-HNE and Ponceau Rouge. Data were normalized to Ponceau Rouge and expressed as relative to control. 4-HNE: 4-hydroxynonenal (*n* = 6/group). (c) Levels of carbonylated proteins, representative result of a membrane incubated with anti-DNPH and anti-*β*-actin using the OxyBlot® Kit. Data were normalized to *β*-actin and expressed as relative to control. DNPH: 2,4-dinitrophenylhydrazine. CTRL: control; 0 h; euthanasia immediately after exercise; 30 min: euthanasia 30 minutes after exercise; 1 h: euthanasia 1 hour after exercise; 2 h: euthanasia 2 hours after exercise. The data were expressed as the mean ± standard error of the mean (*n* = 5/group). ^∗^*p* < 0.05; ^∗∗^*p* < 0,005, ^∗∗∗^*p* < 0.01; ^∗∗∗∗^*p* < 0.0001.

Since we observed an increase in NOX and mitochondrial ROS production accompanied by decreased CAT activity immediately after exercise session, we decided to measure two biomarkers of oxidative damage to indirectly evaluate ROS availability and the direct impact of ROS in biological macromolecules [37]. Based on this, we analyzed three specific markers of biomolecule oxidation: reactive protein thiol, protein carbonyl, and lipid peroxidation levels. The levels of reactive protein thiols were lower in 0 h group when compared to control (CTRL vs. 0 h, *p* < 0.01) and 2 h group (0 h vs. 2 h, *p* < 0.05). No differences were observed among CTRL, 30 min, 1 h, and 2 h groups (Figure 5(a)). These results suggest that exercise elicited a prooxidative environment in WAT immediately after the session (observed by the oxidation of thiol groups) that was followed by a return to baseline levels.

Lipid peroxidation levels were higher in 0 h (CTRL vs. 0 h, *p* < 0.05), 0.5 h group (CTRL vs. 30 min, *p* < 0.05), and 1 h group (CTRL vs. 1 h, *p* < 0.01) when compared to control (Figure 5(b)). Besides that, protein carbonyl levels were higher in 0 h (CTRL vs. 0 h, *p* < 0.01) and 0.5 h. groups (CTRL vs. 30 min, *p* < 0.01) in comparison to control. In 1 h and 2 h groups, protein carbonylation levels have decreased in relation to 0 h (0 h vs. 1 h, *p* < 0.0001; 0 h vs. 2 h, *p* < 0.0001) and 30 min (30 min vs. 1 h, *p* < 0.0001; 30 min vs. 2 h, *p* < 0.0001) (Figure 5(c)). These results are in line with reactive protein thiol levels, suggesting a transient pro-oxidative state after exercise session.

### 3.6. Effects on Antioxidants Gene Expression

**Figure 6:**
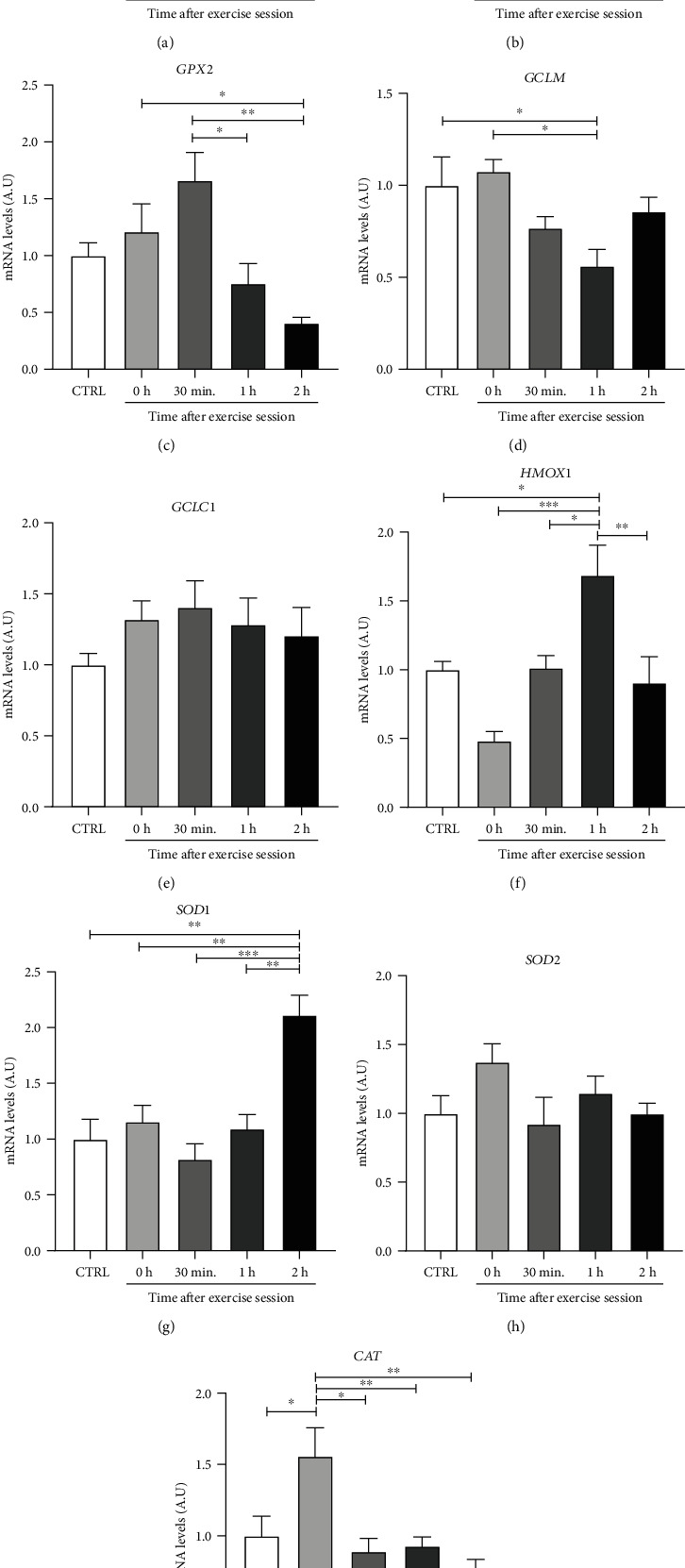
Effect of an acute exercise session on mRNA expression of genes related to antioxidant defense in retroperitoneal WAT. CTRL: control; 0 h; euthanasia immediately after exercise; 30 min: euthanasia 30 minutes after exercise; 1 h: euthanasia 1 hour after exercise; 2 h: euthanasia 2 hours after exercise. (a) NRF2: nuclear erythroid factor related to factor 2; (b) GPX1: glutathione peroxidase 1; (c) GPX2: glutathione peroxidase 2; (d) GCLM: glutamate-cysteine ligase modifying subunits; (e) GCLC1: catalytic subunit 1 glutamate-cysteine ligase; (f) HMOX1: heme oxygenase 1; (g) SOD1: superoxide dismutase 1; (h) SOD2: superoxide dismutase 2; (i) CAT: catalase. Data were expressed as the mean ± standard error of the mean (*n* = 5). ^∗^*p* < 0.05; ^∗∗^*p* < 0.01; ^∗∗∗^*p* < 0.001.

Physical exercise can activate redox-sensitive intracellular signaling pathways through ROS-related mechanisms in several tissues, leading to physiological modifications through both genomic and non-genomic mechanisms [38]. Since we observed that our exercise protocol resulted in increased ROS availability in retroperitoneal WAT, we analyzed if antioxidant and cytoprotective genes related to the activation of NRF2-KEAP1 pathway would be modulated (Figure 6).

One hour after exercise (1 h), a significant increase in NRF2 mRNA levels was observed in relation to the CTRL (*p* < 0.05), 0 h (*p* < 0.001), and 2 h (*p* < 0.05) groups (Figure 6(a)). GPX2 mRNA levels were higher in 30 min group in relation to 1 h (*p* < 0.05) and 2 h (*p* < 0.01) groups (Figure 6(c)). Glutamate-cysteine ligase modifying subunits (GCLM) mRNA levels were lower in 1 h group when compared to CTRL (*p* < 0.05) and 0 h (*p* < 0.05) groups (Figure 6(d)). Heme oxygenase1 1 (HMOX1) mRNA levels were higher in 1 h group in relation to the CTRL (*p* < 0.05), 0 h (*p* < 0.001), 30 min (*p* < 0.05), and 2 h (*p* < 0.01) groups (Figure 6(f)). SOD1 gene expression was higher in 2 h group in comparison to CTRL (*p* < 0.01), 0 h (*p* < 0.01), 30 min (*p* < 001), and 1 h (*p* < 0.01) groups (Figure 6(g)). It was also observed a significant increase in CAT expression in 0 h in relation to CTRL (*p* < 0.05), 30 min (*p* < 0.05), 1 h (*p* < 0.01), and 2 h (*p* < 0.01) groups (Figure 6(i)), thus suggesting that the reduction of catalase activity after exercise (Figure 5(a)) was due to posttranslational mechanisms. There were no significant changes of SOD2, Catalytic Subunit 1 Glutamate-Cysteine Ligase (GCLC1) and GPX1 mRNA levels in any of the postexercise periods evaluated. These results show that one acute aerobic exercise session was able to modulate the mRNA levels of some antioxidant and cytoprotective genes.

## 4. Discussion

Several studies have shown that chronic exposure to exercise results in enhanced cytoprotection and antioxidant defenses in different tissues, such as skeletal muscle [10], heart [39], brain [40], and others [41]. This physiological adaptation is related to the induction of low levels of ROS in each single bout of exercise [38]. In WAT, there are several pieces of evidence showing that chronic exposure to aerobic exercise is linked to lipid storage reduction, fatty acid mobilization [42], improved mitochondrial function [43], decreased expression of inflammatory adipokines [20], and consequently modified WAT metabolism [44], and phenotype [45]. Chronic exercise seems to elicit redox adaptations in WAT, such as decreased ROS production and increased antioxidant defense [46]. Our study revealed that acute moderate-high intensity endurance exercise promoted a transient prooxidative state in WAT, which resulted in transient oxidation of biomolecules, and increased antioxidant/cytoprotective gene expression.

In the present study, we evaluated two important sources of ROS in WAT, NOX enzymes and mitochondria [47, 48]. NOXs are enzymes whose only function is the production of superoxide or H_2_O_2_ [49]. They are transmembrane enzymes and belong to the NOX family, including NOXs from 1 to 5, and DUOXs 1 and 2, which show different tissue distribution and expression levels [50]. NOX2 and NOX4 seem to be the most expressed NOX isoforms in WAT [51], but we could not find any evidence in the literature about the effect of acute exercise on their activities. In the present work, we observed a transient increase in NOX-derived ROS production, which was higher immediately after the exercise session and also 30 minutes later, then returning to baseline levels. NOX enzymes can be activated by several factors that are increased during acute exercise, which was not the aim of the present study.

The increase of mitochondrial respiration is associated with the electron escape of complex I and III, leading to a partial reduction of O_2_ forming superoxide anion (O_2_^•−^) [52], which increases extramitochondrial H_2_O_2_ efflux [52]. Although we have found an increase in mitochondrial respiration immediately after exercise, ATP production was reduced. Moreover, an inverse relationship between ATP and ROS production was also observed. Das and Jana (2015) observed higher levels of protein carbonyls in the *α*-subunit of the F1 complex of skeletal muscle ATP synthase of old mice when compared to their young counterparts. Interestingly, it was associated with a lower activity of this channel in old animals [53]. This is strong evidence of redox-sensitive sites at ATP synthase, and that redox modifications of the ATP synthase *α*-subunit could play an important role in the regulation of ATP synthesis [54]. Wang et al. (2011) demonstrated in mitochondria isolated from rat hearts that blocking the oxidation of free thiols with N-Ethylmaleimide (NEM, an alkylating reagent) led to increased ATPase enzyme activity. The authors have shown that the difference between the groups treated and not treated with NEM was large (two times), suggesting that the oxidative modification of Cys have a profound effect on the ATPase activity. Still in this study, the authors observed the presence of a disulfide bond between the ATP synthase *α*-subunit and the *γ*-subunit. Nevertheless, the ATP*α* subunit was identified as the main mitochondrial protein that undergoes oxidative changes induced by ROS, which is associated with reduced activity of the enzyme ATPase [55]. In our findings, we observed that ATP production was reduced immediately after exercise, at the same moment that ROS levels increased. However, from 30 minutes after exercise, ATP production increased, indicating a possible mechanism of ROS modulating mitochondrial ATP production.

ROS availability in a given tissue depends on their production and detoxification. Since our exercise session increased NOX- and mitochondria-derived ROS production, we investigated the activity of the antioxidant enzymes SOD, CAT, and GPX. Nevertheless, no differences among groups were observed concerning SOD and GPx activities. On the other hand, we observed a significant reduction in CAT activity at all-time points after the exercise session. Ping et al. (2015) showed in male mice skeletal muscle a similar response to acute aerobic exercise, with an increase in mitochondrial H_2_O_2_ production, a reduction in CAT activity up to 90 minutes after exercise, and no differences in SOD activity [56]. CAT is a peroxisomal enzyme that converts H_2_O_2_ into water and O_2_ [57]. Since catalase detoxifies H_2_O_2_, the decrease in its activity observed after exercise may result in greater availability of ROS.

Our results showed that reactive protein thiol levels were significantly lower immediately after exercise. However, these effects were transient, returning to baseline levels 2 hours after exercise, indicating a recovery from the prooxidant state. Thiols include any organosulfur compound containing the group R-SH in the reduced state (R represents an alkyl group or other organic substituent). The oxidation of thiol groups in cysteine residues often leads to the formation of disulfide bridges, which can importantly impact the tridimensional structure and activity of a protein, for example [58]. Reduced cysteine thiol (Cys-SH) and their oxidized disulfide counterparts are carefully balanced to maintain redox homeostasis in various cellular compartments. Because of that, their levels are related to ROS availability. Moreover, there is a consistent body of evidence showing that transient thiol oxidation is related to redox-sensitive signaling modulation, impacting on cell-signaling proteins, modifying protein kinase activity, and transcription factors [59, 60].

We also observed an increase of lipid peroxidation right after the exercise session, which was sustained after 0.5 and 1 h. Previous reports demonstrated an increase of lipid peroxidation products in the blood after physical exercise [57]. 4-HNE can react with the thiol and amino groups of macromolecules and it is linked to the formation of stable covalent Michael and Schiff base adducts to a wide group of proteins, resulting in protein crosslinking, protein aggregation, inactivation of protein function, and structural perturbation, including increase protein carbonylation [58–60]. Moreover, transient increases in 4-HNE levels have been associated with the activation of Nrf2, increasing mitochondriogenesis, and antioxidant defense [61].

So next, we also evaluated protein carbonyl levels, another marker of oxidation that is formed due to oxidative deamination of lysine and glutamic acid [57]. We observed an increase in protein carbonyl levels immediately and 30 minutes after the end of the exercise session, which returned to basal levels after one hour. These results are in line with the pattern of the most of redox parameters assessed here (increased ROS generation, decreased CAT activity, reduced protein thiol content, and increased 4-HNE) observed immediately after exercise, showing that acute exercise elicited a transitory pro-oxidative state in WAT.

Aerobic exercise in rodent models has consistently been shown to activate Nrf2 signaling in multiple tissues, including skeletal muscle [59], liver [62], and myocardium [63], which leads to the upregulation of endogenous antioxidant gene and protein expressions, one of the physiological adaptations related to the beneficial effect of physical exercise [7]. In basal conditions, NRF2 remains located in the cytoplasm, linked to Kelch-like ECH-associated protein 1 (Keap1), being inactive. The association with Keap1 leads to ubiquitination and, consequently, protease-mediated degradation of NRF2. However, an increase of ROS availability can trigger Keap1 oxidation, decreasing NRF2-Keap1 interaction and, consequently, reducing NRF2 degradation [64]. This process increases NRF2 translocation to the nucleus and its binding to antioxidant response element (ARE), leading to the transcription of more than 200 cytoprotective genes, including NRF2 itself [7]. We observed an increase of NRF2 mRNA levels 1 hour after the exercise session, which suggests that NRF2 signaling was activated in our model, probably due to the transient increase in ROS generation.

Furthermore, one session of exercise was also able to increase mRNA levels of several antioxidant and cytoprotective genes related to NRF2 activation in WAT, including HMOX, SOD1, CAT, and GCLM. The mRNA levels of HMOX1 were higher in the 1 h group. HMOX1 gene codifies hemeoxygenase 1, the enzyme that catalyzes the first and rate-limiting step in heme degradation reaction, producing CO, iron, and biliverdin. HMOX1 is involved in antioxidant anti-inflammatory functions, and it is present at very low levels in most cells and tissues, being upregulated in prooxidant conditions [65]. SOD1 gene expression was also increased after exercise. This enzyme is one of three SOD isoforms, responsible for the dismutation of O_2_^•−^ to H_2_O_2_. CAT gene expression increased immediately after exercise; however, it returned to basal level 30 minutes after the session. CAT gene encodes catalase, a key antioxidant enzyme present in the peroxisome of nearly all aerobic cells that converts H_2_O_2_ to water and oxygen [66]. Curiously, an acute reduction in GCLM gene expression was lower in the 1 h group in comparison to CTRL and 0 h groups. GCLM is related to the glutathione synthesis from L-cysteine and L-glutamate [67]. The lack of response regarding the expression of GPX1, GPX2, GCLC1, GCLM, and SOD2 was inconsistent with the increase found in other antioxidant genes. The literature is scarce in the description of the effect of acute physical exercise on the dynamics of regulation of cytoprotective genes. Thus, more studies are necessary to elucidate this question.

In the present study, the dynamics of DNA oxidation and DNA damage response after acute exercise were not evaluated, but this is an important topic that will be addressed in future studies. Moreover, the effect of chronic aerobic exercise on WAT redox homeostasis and DNA damage response of control and obese animals is also a relevant issue that our group intends to evaluate.

## 5. Conclusion

In conclusion, we demonstrate that one session of aerobic exercise induced a transient prooxidant environment, characterized by a higher NOX activity and extramitochondrial ROS production, decreased CAT activity, and higher levels of biomolecules oxidation. Moreover, mRNA levels of antioxidant and cytoprotective genes were increased by acute exercise, suggesting a cellular response to the transient ROS exposure.

## Figures and Tables

**Table 1:** Description of primers used for real-time PCR.

GENE	Forward sequence	Reverse sequence
CAT	CAAGCTGGTTAATGCGAATGG	TTGAAAAGATCTCGGAGGCC
NFE2L2	TTTGTAGATGACCATGAGTCGC	TGTCCTGCTGTATGCTGCTT
HMOX1	ATCGTGCTCGCATGAACACT	CAGCTCCTCAAACAGCTCAATG
GCLM	CAGTGGGCACAGGTAAAACC	AATGCAGTCAAATCTGGTGGC
GPX1	AATCAGTTCGGACATCAGGAG	GAAGGTAAAGAGCGGGTGAG
GPX2	ACCGATCCCAAGCTCATCAT	TCTCAAAGTTCCAGGACACATCTG
SOD1	TGTGTCCATTGAAGATCGTGTG	CTTCCAGCATTTCCAGTCTTTG
SOD2	GGACAAACCTGAGCCCTAAG	CAAAAGACCCAAAGTCACGC
GCLC1	GGTGACGAGGTGGAGTACAT	AACATCGCCGCCATTCAGTA

## Data Availability

All data used to support the findings of this study are available from the corresponding author upon request.
